# Supplementary material for: 4D imaging of lithium-batteries using correlative neutron and X-ray tomography with a virtual unrolling technique
Source: Nat Commun. 2020 Feb 7;11:777. doi: 10.1038/s41467-019-13943-3 (PMC7005889; doi:10.1038/s41467-019-13943-3)
Supplement: Supplementary file 1 — Supplementary Information [file 41467_2019_13943_MOESM1_ESM.pdf]

Supplementary Information:

# 4D imaging of Li-batteries using correlative neutron and X-ray tomography with a virtual unrolling technique

Ziesche et al.

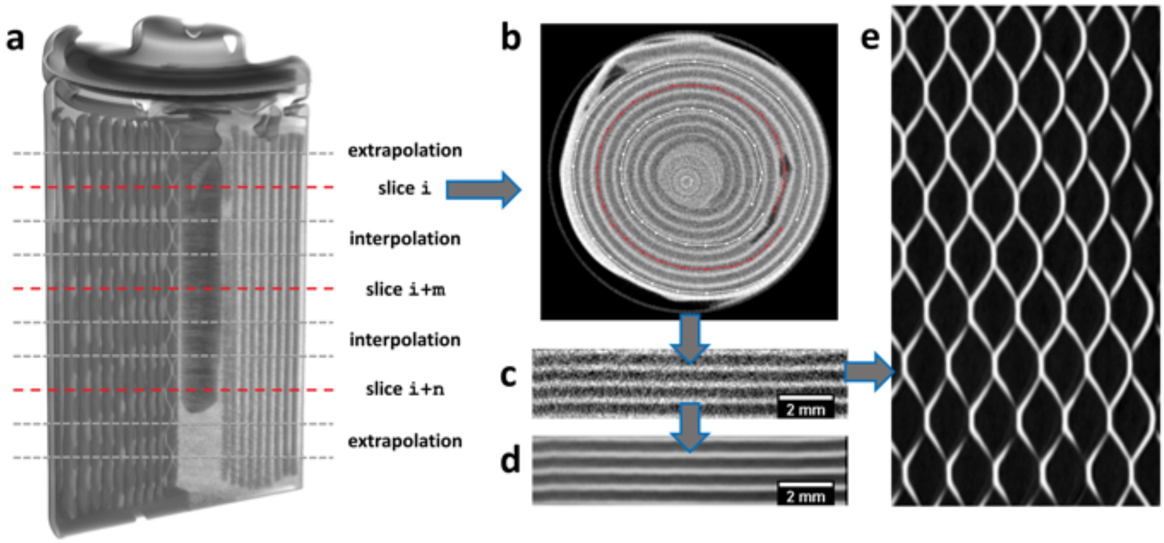


**Supplementary Figure 1:** **Working principle of unfolding process:** a) 3D volume of the battery. Slices `i`, `i+m` and `i+n` are defined by the experimentalist. b) Cross-section of slice `i` with contour. c) Unrolled slice along contour which is depicted in b). d) Averaging same line profiles taken from several slices increases the signal-noise-ratio. e) The mesh of the current collector can be extracted as a flattened 3D volume.

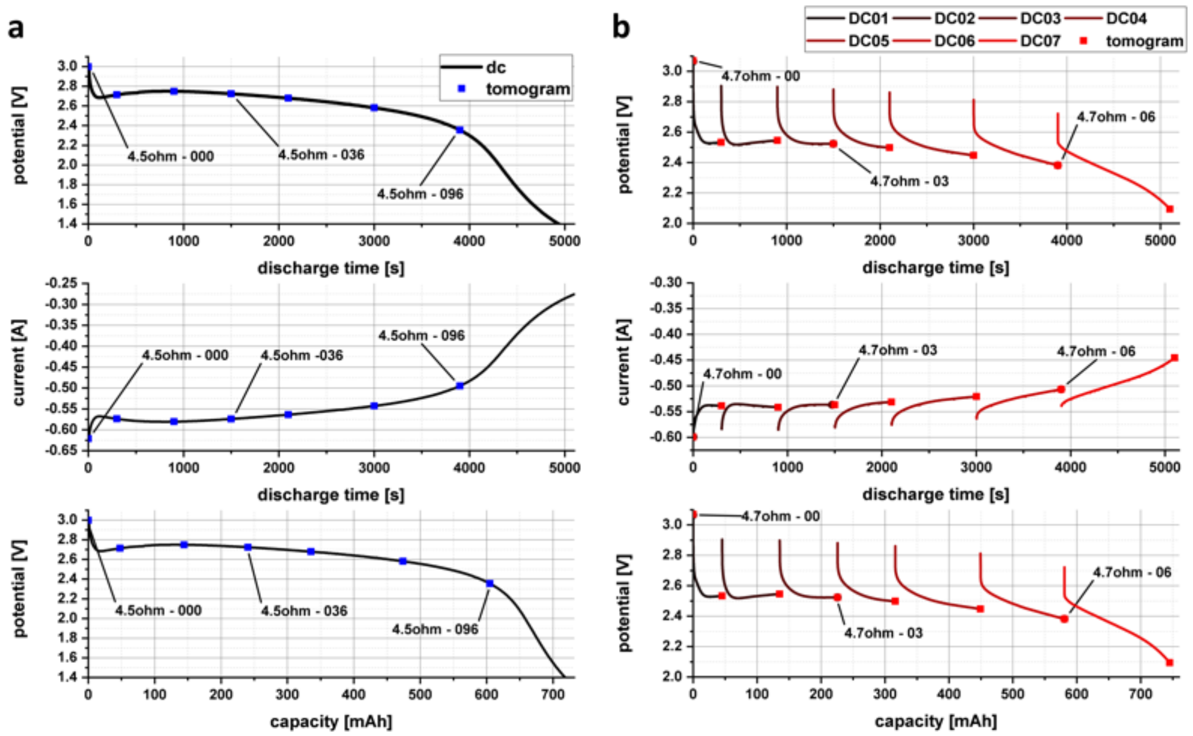


**Supplementary Figure 2:** **CR2 discharge profiles for the X-ray and neutron experiment:** a) the graphs show the constant resistance discharge curves for the CR2 cell over 4.5 Ω, where simultaneous fast X-ray CT was carried out. The graphs in b) show the discharge curve over 4.7 Ω, where the discharge was interrupted for each neutron tomography after a certain amount of time. The plots from top to bottom display the cell potential and current as a function of time and capacity. The blue and red boxes mark the discharge parameters for which tomograms were recorded.

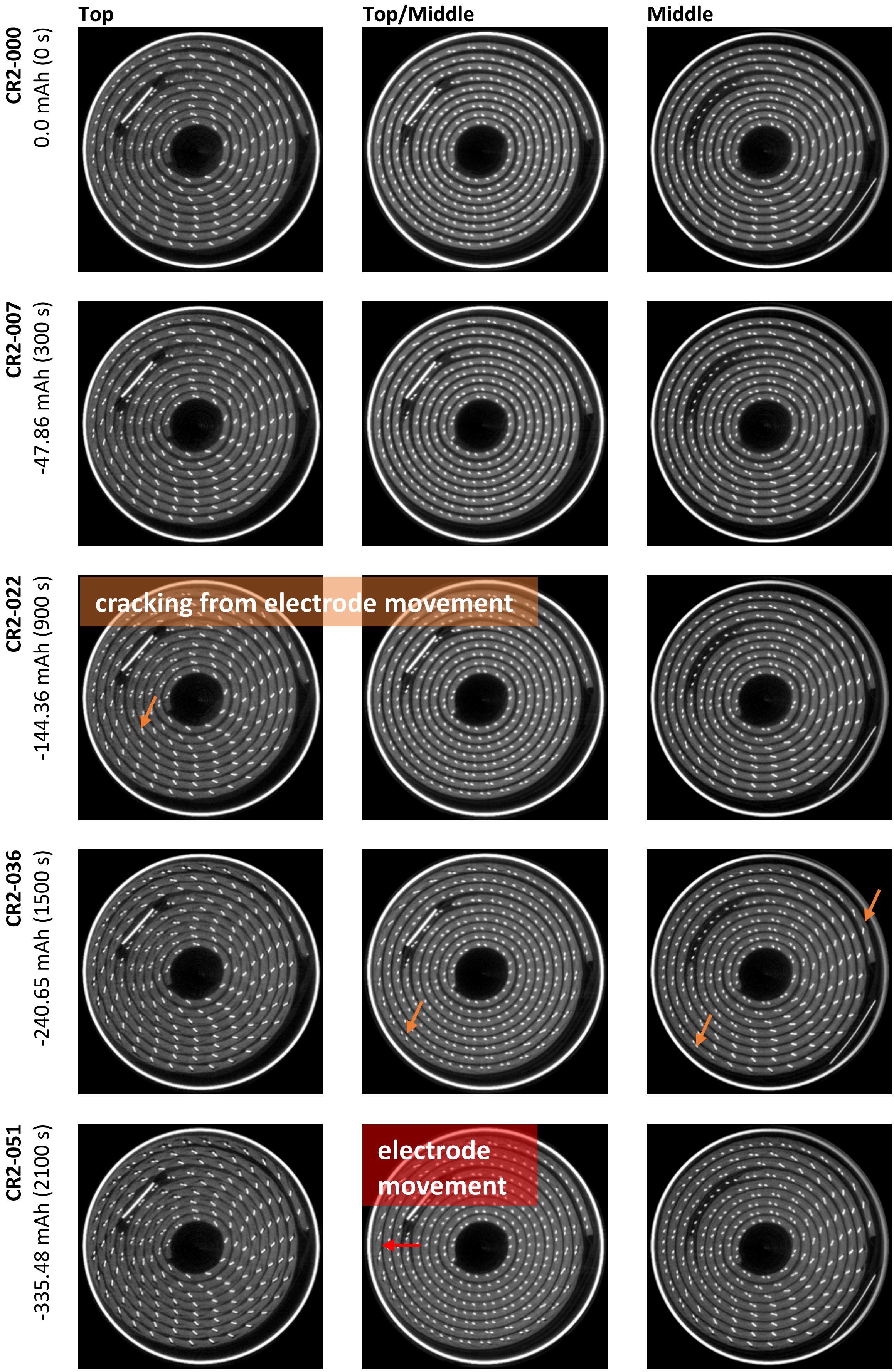
Top
Top/Middle
Middle
CR2-000
0.0 mAh (0 s)
CR2-007
-47.86 mAh (300 s)
CR2-022
-144.36 mAh (900 s)
cracking from electrode movement
CR2-036
-240.65 mAh (1500 s)
CR2-051
-335.48 mAh (2100 s)
electrode movement

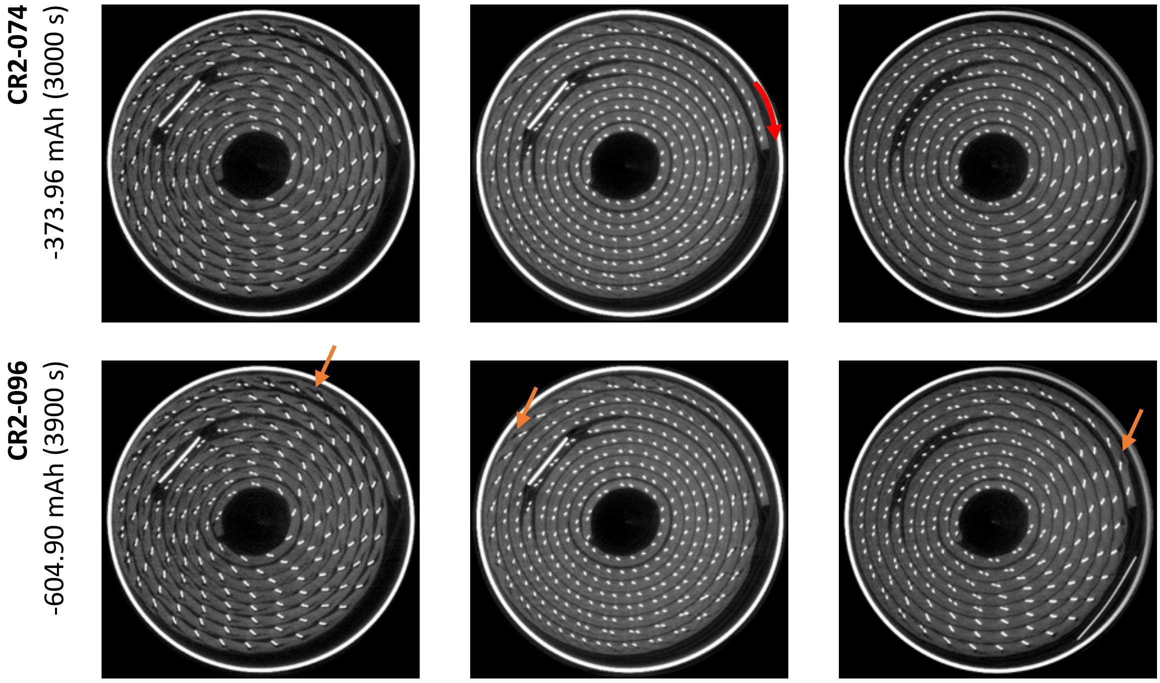


**Supplementary Figure 3: Horizontal cross sections through the X-ray tomograms at different SoC and heights:** The images show horizontal slices out of the X-ray tomograms at different cell heights from the top, top/middle and the middle part, at different SoC. There are 103 tomograms in total, labelled from CR2-000 to CR2-102. One tomogram was recorded every 40 s with a total acquisition period of 2.8 s. For the analysis, tomograms were used with an expanding time interval between their recording time due to the fast structural changes in the first time period of the discharge. The image contrast was optimised for the lower attenuating components in order to improve the visibility of the structural changes in the lower attenuating cathode material. Arrows highlight, as examples, the $MnO_2$ electrode cracking in orange and the electrode movement in red.

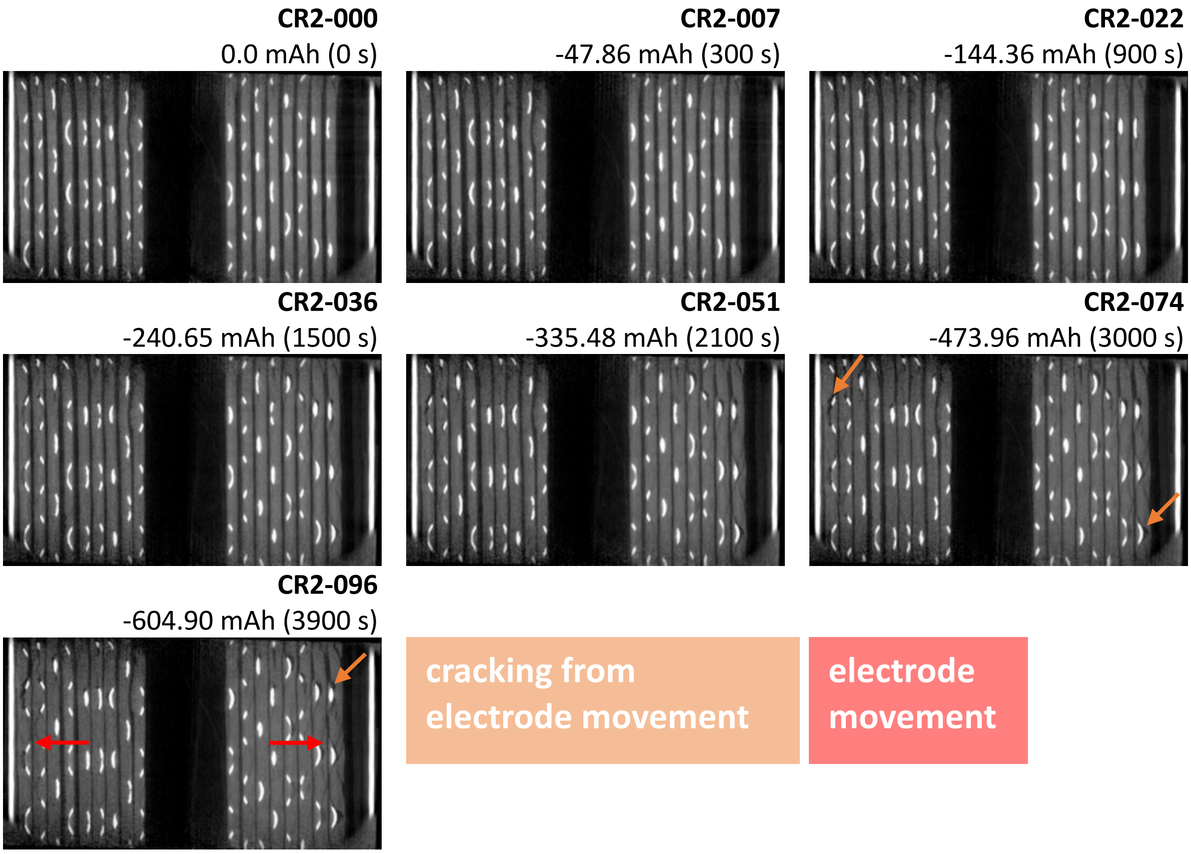


**Supplementary Figure 4:** **Vertical cross sections through the X-ray tomograms at different SoC:** The images show vertical slices from the X-ray tomograms at different SoC. In total, there are 103 tomograms made labelled from CR2-000 to CR2-102. One tomogram was collected every 40 s with a total acquisition period of 2.8 s. For the analysis, tomograms were used with an expanding time interval between their recording time due to the fast structural changes in the first time period of the discharge. The image contrast was optimised for the lower attenuating components in order to improve the visibility of the structural changes in the lower attenuating cathode material. Arrows highlight, as examples, the $MnO_2$ electrode cracking in orange and the electrode movement in red.

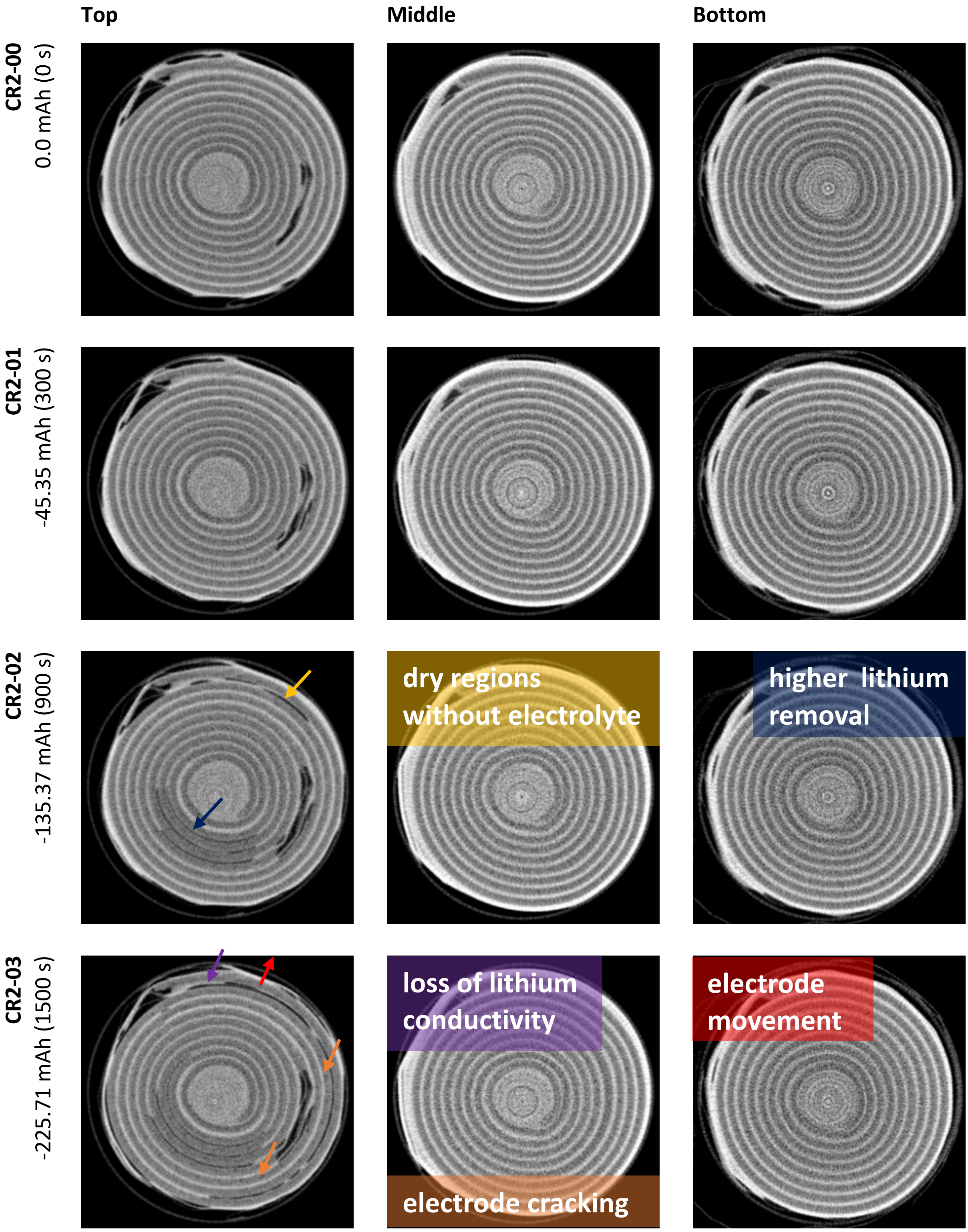
Top
Middle
Bottom
CR2-00
0.0 mAh (0 s)
CR2-01
-45.35 mAh (300 s)
CR2-02
-135.37 mAh (900 s)
dry regions without electrolyte
higher lithium removal
CR2-03
-225.71 mAh (1500 s)
loss of lithium conductivity
electrode cracking
electrode movement

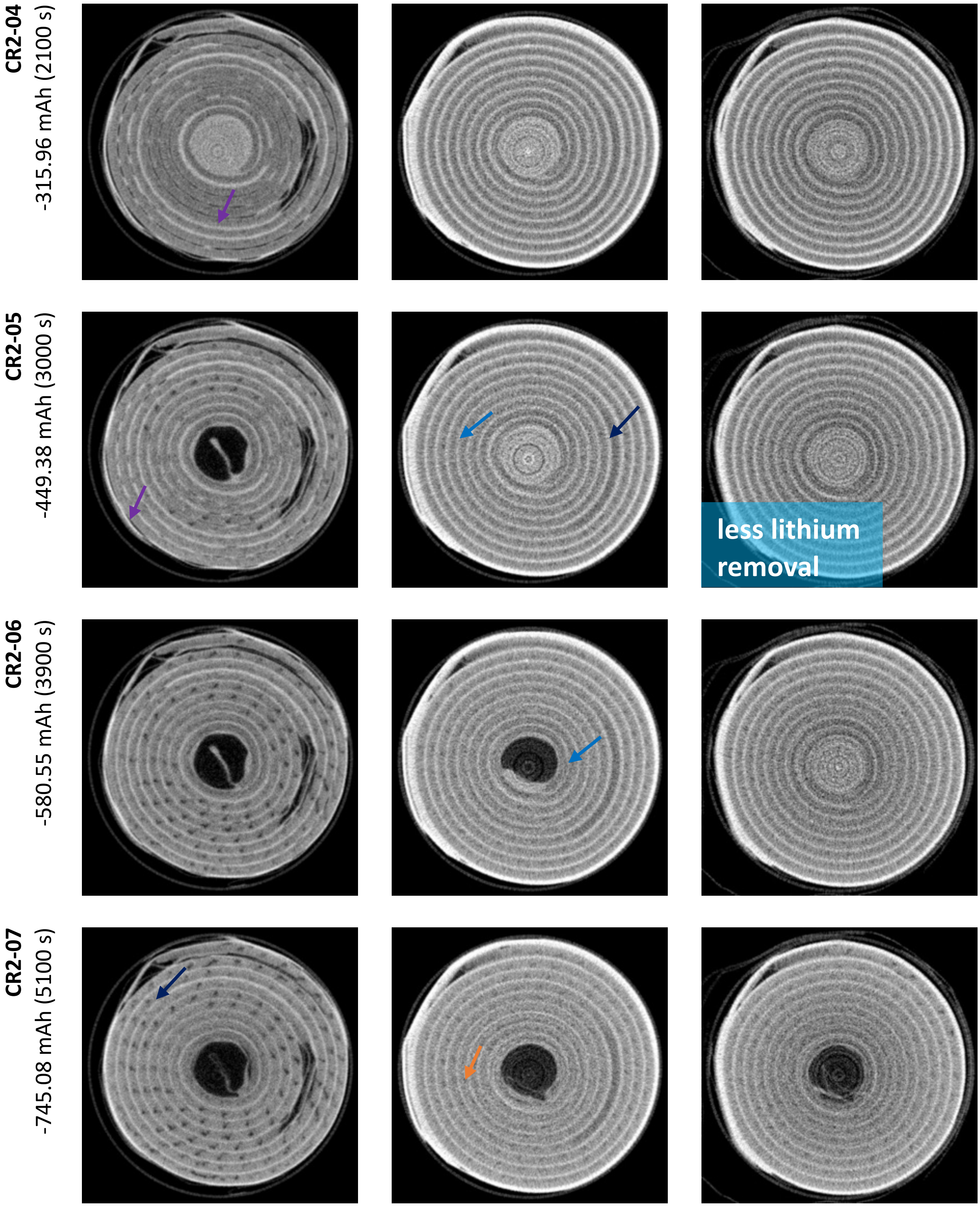


**Supplementary Figure 5: Horizontal cross sections through the neutron tomograms at different SoC and heights:** The images show horizontal slices of the neutron tomograms captured during medium c-rate discharge. In total, eight neutron tomograms were recorded with an acquisition period of about 8 h. The discharging process was interrupted for each tomogram and labelled with CR-00 from the pristine to CR2-07, the partly discharged SoC of -745.08 mAh. Arrows highlight, as examples, the loss of lithium conductivity in purple and areas of lower and higher lithium removal from the lithium metal electrode in bright and dark blue.

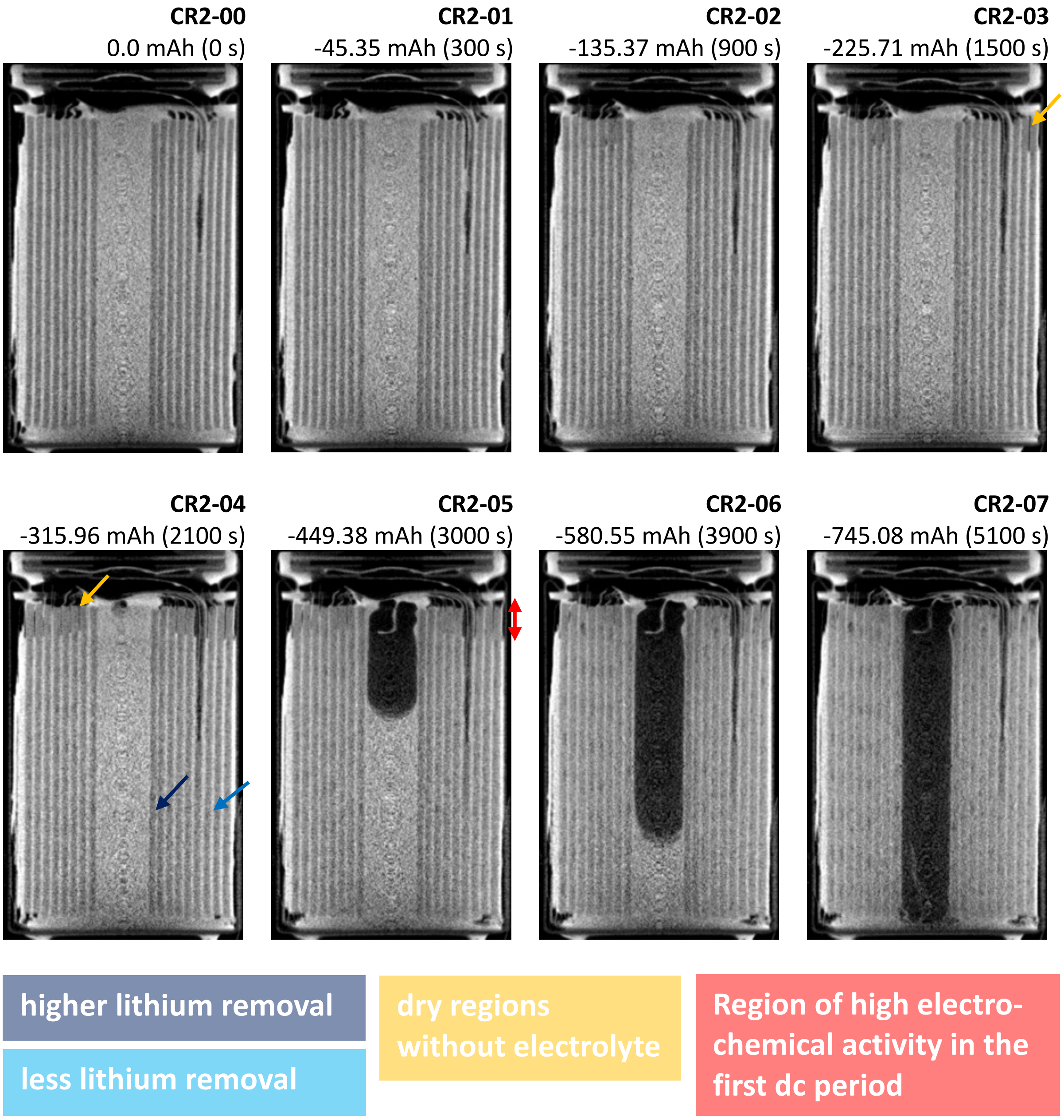


**Supplementary Figure 6: Vertical cross sections through the neutron tomograms at different SoC:** The images show vertical slices of the neutron tomograms captured during medium c-rate discharge. In total, eight neutron tomograms were made with an acquisition period of about 8 h. The discharging process was interrupted for each tomogram and labelled with CR-00 from the pristine to CR2-07, the partly discharged SoC of -745.08 mAh. Arrows highlight, as examples, the loss of lithium conductivity in purple and areas of lower and higher lithium removal from the lithium metal electrode in bright and dark blue.

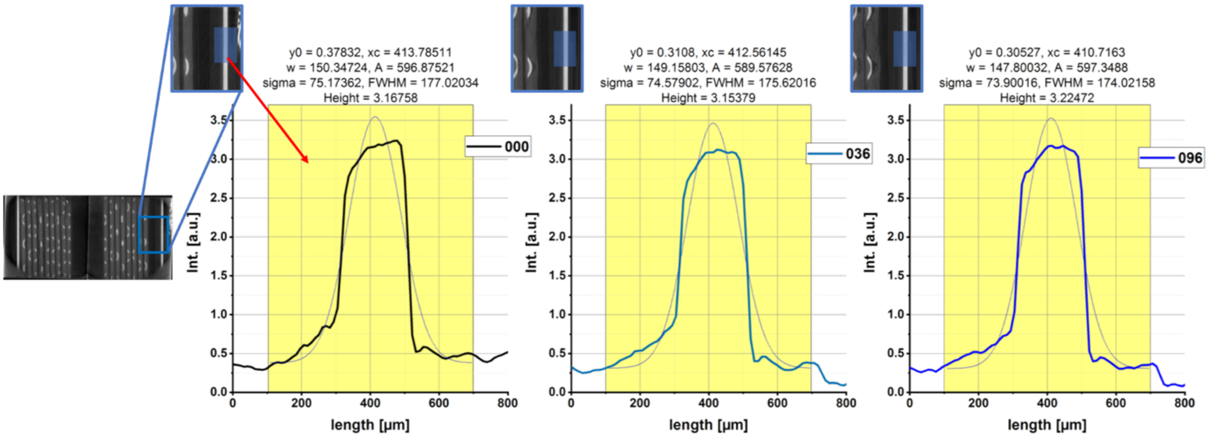


**Supplementary Figure 7: Error calculation of the thickness deviation for the different SoC of the X-ray tomographies:** Error calculation by using the intensity variation of the cell casing during the discharging process. As reference the intensity profile of a piece of the casing out of a vertical orthogonal slice was used and for a better statistic the profile was integrated over about 100 pixel lines. The thickness was then determined by using the FWHM of the intensity plot. Here shown for the X-ray data, the calculated error between the measurements show a deviation smaller as 1.5 µm. The average casing thickness, over the discharge, was determined to be 175.5 µm.

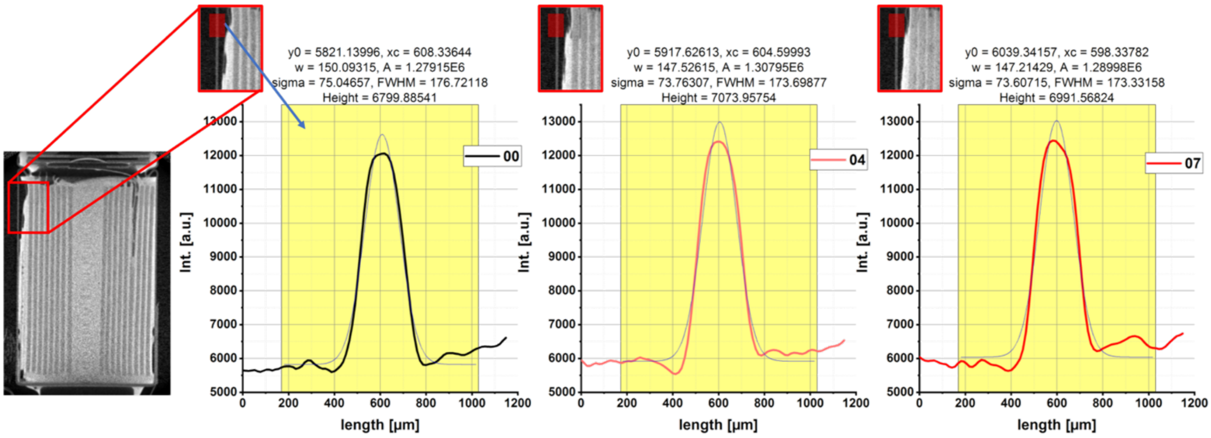


**Supplementary Figure 8: Error calculation of the thickness deviation for the different SoC of the Neutron tomographies:** Error calculation by using the intensity variation of the cell casing during the discharging process. As reference the intensity profile of a piece of the casing out of a vertical orthogonal slice was used and for a better statistic the profile was integrated over about 100 pixel lines. The thickness was then determined by using the FWHM of the intensity plot. Here shown for the neutron data, the calculated error between the measurements show a deviation smaller as 1.5 µm. The average casing thickness, over the discharge, was determined to be 174.6 µm.
